# A cross-sectional prospective study of seclusion, restraint and involuntary medication in acute psychiatric wards: patient, staff and ward characteristics

**DOI:** 10.1186/1472-6963-10-89

**Published:** 2010-04-06

**Authors:** Tonje Lossius Husum, Johan Håkon Bjørngaard, Arnstein Finset, Torleif Ruud

**Affiliations:** 1 SINTEF Health Services Research, PB 124, 0314 Oslo, Norway; 2 Norwegian University of Science and Technology (NTNU), Faculty of Medicine, Department of Public Health and General Practice, Trondheim, Norway; 3 University of Oslo, Faculty of Medicine, Oslo, Norway; 4 Akershus University Hospital, Division of Mental Health Services, Akershus, Norway; 5 University of Oslo, Faculty of Medicine, Institute of Psychiatry, Oslo, Norway

## Abstract

**Background:**

Previous research on mental health care has shown considerable differences in use of seclusion, restraint and involuntary medication among different wards and geographical areas. This study investigates to what extent use of seclusion, restraint and involuntary medication for involuntary admitted patients in Norwegian acute psychiatric wards is associated with patient, staff and ward characteristics. The study includes data from 32 acute psychiatric wards.

**Methods:**

Multilevel logistic regression using Stata was applied with data from 1016 involuntary admitted patients that were linked to data about wards. The sample comprised two hierarchical levels (patients and wards) and the dependent variables had two values (0 = no use and 1 = use). Coercive measures were defined as use of seclusion, restraint and involuntary depot medication during hospitalization.

**Results:**

The total number of involuntary admitted patients was 1214 (35% of total sample). The percentage of patients who were exposed to coercive measures ranged from 0-88% across wards. Of the involuntary admitted patients, 424 (35%) had been secluded, 117 (10%) had been restrained and 113 (9%) had received involuntary depot medication at discharge. Data from 1016 patients could be linked in the multilevel analysis. There was a substantial between-ward variance in the use of coercive measures; however, this was influenced to some extent by compositional differences across wards, especially for the use of restraint.

**Conclusions:**

The substantial between-ward variance, even when adjusting for patients' individual psychopathology, indicates that ward factors influence the use of seclusion, restraint and involuntary medication and that some wards have the potential for quality improvement. Hence, interventions to reduce the use of seclusion, restraint and involuntary medication should take into account organizational and environmental factors.

## Background

Use of coercion in treatment is controversial [1-5], and reducing use of coercion in psychiatric services is a priority health political issue in Western countries [6-8]. Too much use of coercion in mental health care may be a threat to the quality of care, as well as to patients' human rights. It is of crucial importance to develop a better understanding of the processes and factors involved to reduce the use of coercion. There is evidence of considerable variation in the extent to which coercive measures are used. This is shown in international comparative studies [9-11], and among wards and geographical areas in the same country [12-21]. A recent literature review of the incidence of seclusion and restraint comparing data from 12 countries concludes that available data suggest there are major differences among them in the percentage of patients subjected to coercion and the duration of coercive interventions [22]. Several hypotheses are put forward on factors that may explain differences in coercion. These factors can be divided into four groups [23]. The list is not exhaustive and some factors may belong to several categories.

### Structural factors

Physical characteristics of ward, size of ward, double or single rooms, crowding and patient turnover [12,13,18,21,24-26].

### Staff-related factors

Staff/patient ratio, age and sex of staff, experience of staff, proportion of unqualified staff, level of qualifications, de-escalation training, staff turnover, attitudes of staff and administrators [12,13,16,27-39].

### Patient-related factors

Diagnoses, level of aggression, symptoms, age and sex, ethnicity, time of day, season [12,18,20,21,32,40].

### Treatment-related factors

Pharmacological treatment, use of psychotherapy, treatment by staff including limit setting, activities for patients, ward atmosphere, treatment philosophy and ideology, regulations and guidelines on use of restraint and seclusion, transitions in ward routines [1,12,28,29,34,35,37].

Taken together, the results from studies on differences in the use of coercive measures are not conclusive. Studies tend to be small, and there are few larger comparative studies. A key question is whether differences in the use of coercion among wards may be attributed mainly to composite differences in patient characteristics or to contextual effects such as ward culture, organization or staff attitudes. Our study investigates both patient and ward factors as possible predictors of differences in the use of coercion, and it is to our knowledge the first such study using a statistical multilevel approach.

The aims of the study are to:

(i) investigate frequency and variance in the use of coercive measures in acute psychiatric wards in Norway, and (ii) identify predictors of the use of coercion for involuntary admitted patients, with emphasis on patient, staff and ward characteristics, investigating especially whether mean ward-level staff attitudes to coercion influence the use of coercion.

## Methods

### Design and sample

The study was part of the Multicenter study of Acute Psychiatry (MAP) in Norway in 2005-2006, which was carried out by an acute mental health services research network as a cross-sectional prospective study [41]. It was possible to link data about wards with data about patients from 32 acute psychiatric wards located in 17 of the 23 acute psychiatric departments across all 5 health regions in Norway. The sample is considered to be representative of Norwegian acute psychiatric wards. Patients were included in the study over a period of 3 months, and data were collected at admission, during hospitalization and at discharge. Data collection was ended after 2 months if the patient had not been discharged during that time. Most patients were, however, discharged before this. Very few patients may have had more than one admission in the 3-month inclusion period. At ward level, data were collected on number of beds, staffing, staff characteristics and attitudes towards coercion. The research institute SINTEF Health Research in Norway organized the network and coordinated the study with support from the Norwegian Directorate of Health and Social Affairs. The study was approved by the Regional Committee for Ethics in Medical Research and by the Privacy Ombudsman on behalf of the Data Inspectorate. The Regional Committee for Ethics in Medial Research approved the study without requiring consent from the patients; thus, data were restricted to chart data only. The sample consisted of 3572 patients and we estimate this to be approximately 95% of patients admitted in the 3-month inclusion period. Of these, 1214 patients were admitted involuntarily. Coercive measures are used almost exclusively with involuntary admitted patients. Hence, voluntary admitted patients were excluded from the multilevel analyses. For the multilevel analysis, it was possible to link data on patients and wards for 1016 involuntary admitted patients.

### Definition of seclusion, restraint and involuntary medication (dependent variables)

Different national legislation and practices in use of coercive measures during treatment are challenges in comparative studies [13,28,42]. Coercive measures during hospitalization in this study are defined as seclusion, restraint and involuntary medication. Data about coercive measures were recorded on registration forms by clinicians and experienced psychiatric nurses in the teams treating patients. This was done at the end of the stay when the treatment and use of coercion throughout the whole stay was known. At discharge, staff recorded whether the patient had been subjected to any of these measures during the admission. Use of coercive measures was recorded only with yes or no and not with number of times or duration. The use of a coercive measure requires specific decisions that are written in patient records. These records were considered to be highly accurate data that did not require additional tests of validity or reliability.

#### Seclusion

The practice of seclusion in Norway resembles the concepts of "open area seclusion", "segregation nursing", "segregation area", "quiet rooms" or "sheltered area" in international literature (28). The word "shielding" has also been used. However, there is some variation in the Norwegian use of the concept. The seclusion area can range from a single room to small, separate units or areas inside wards [43]. Norwegian mental health law requires that patients in seclusion should not be left alone and should be accompanied by staff. However, research in Norway on seclusion has shown that patients may experience this practice as resembling the more common international use of seclusion, which in Norway is called isolation [43]. For this reason, we have chosen to use the term "seclusion" in this article, and we define it as confining a patient in a single room or in a separate unit or area inside the ward, accompanied by staff.

#### Restraint

Restraint is defined as strapping a patient to a bed with mechanical devices (belts). In Norway, bed belts with 5-point restraints are used. This is a bed with belts over the patient's arms, legs and torso. Not all belts need to be used at all times.

#### Involuntary medication

In Norway, legislation differentiates between the involuntary admission itself and involuntary treatment during the stay, which is not the case in many other countries. There is also a distinction between involuntary medication as a treatment intervention and involuntary medication as an acute intervention in crisis. In this study, we used a variable to indicate whether the patient was involuntarily treated with depot medication at discharge. Depot medication is used at this point as treatment and not as a chemical restraint in an acute crisis, which seldom happens in Norway and is not included in this study. Not all countries have this distinction, which may make comparison across studies difficult.

### Patient level variables

Patients diagnosed with schizophrenia or psychosis (F20-F29 in ICD-10) [44] were compared with patients with other diagnoses. The severity of mental health problems was measured at admission using the Health of the Nation Outcome Scales (HoNOS), with 12 items covering various key problem areas for patients with severe mental illness [45], and also clinical and social functioning. Each problem area is rated on a scale from 0 to 4, with higher ratings for more severe problems. The scoring of HoNOS was done by clinicians and experienced psychiatric nurses on the team treating the patient. The raters were trained in HoNOS in a half-day session with instruction about HoNOS, discussion of each scale and training on cases followed by discussion of differences in ratings. The design of the training was based on the training model used in the United Kingdom (UK), after a visit from the person in charge of the UK national training programme. Testing of interrater reliability was not done, as it was difficult to engage all clinicians in such procedures, in addition to data collection for the study together with the pressure of their daily clinical work in the acute wards. However, testing of interrater reliability in Norway after similar training has shown acceptable interrater reliability for all HoNOS scales except 8, 11 and 12. This is in agreement with reviews of interrater reliability of HoNOS [46]. The first 7 problem areas were chosen for analyses in this paper: overactive or aggressive behaviour (HoNOS 1), non-accidental self-injury and suicide attempt (HoNOS 2), problem drinking or drug taking (HoNOS 3), cognitive problems (HoNOS 4), physical illness or disability problems (HoNOS 5), hallucinations and delusions (HoNOS 6) and depressed mood (HoNOS 7). Patient characteristics for the whole sample and for involuntary admitted patients are presented in Table 1.

**Table 1:** Sample characteristics of total sample and involuntary admitted patients

Patient variables:	Total sample: 3462 (100%)	Involuntary adm: 1214 (35%)
Mean age (SD)	40 (SD = 15.5)	40 (SD = 16.7)

Sex (female/male) % in brackets	1710/1752 (49/50)	587/625 (48/52)

Norwegian background	3077 (89%)	1053 (88%)

Not Norwegian background	350 (10%)	144 (12%)

Not having own home	715 (21%)	305 (25%)

Previous contact with MH services	2572 (74%)	864 (72%)

GAFS at admission (mean, SD)^a^	36 (12)	31 (11)

GAFF at admission (mean, SD)^a^	38 (11)	34 (11)

F 20-29 diagnosis (ICD-10)	831 (24%)	460 (41%)

Health of the Nation Outcome Scales	mean (SD)	mean (SD)

HoNOS 1 (overactive & aggressive)^b^	.96 (1.23)	1.47 (1.37)

HoNOS 2 (self-injury & suicidal)	.96 (1.35)	.77 (1.30)

HoNOS 3 (drinking & drugs)	1.09 (1.45)	1.02 (1.45)

HoNOS 4 (cognitive problems)	.91 (1.13)	1.24 (1.29)

HoNOS 5 (physical illness & disability)	.67 (1.08)	.65 (1.07)

HoNOS 6 (hallucinations & delusions)	1.35 (1.44)	2.02 (1.47)

HoNOS 7 (depressed mood)	1.65 (1.23)	1.25 (1.26)

^a ^Global Assessment of Function, Scale from 0 to 100 with lower ratings for more severe problems

^b^Scale from 0 to 4 with higher ratings for more severe problems

### Ward level variables

The sample consisted of multidisciplinary staff groups in 37 psychiatric acute wards. Because of problems with linking data from 5 wards, 32 wards were included in the multilevel analyses. Four of the wards were categorized as "admission wards". They were organized as short-term admission and assessment wards with stays limited to 1-2 days. The other wards were traditional acute wards. Mean ward values from 529 individual staff members' attitudes to coercion are included, with a median of 22 staff members (range 3-66) per ward. Estimates based on staff full-time equivalents indicate that approximately 60% of staff members completed questionnaires. More information about the staff groups is presented in a previous article (Husum, Bjørngaard, Finset & Ruud, Staff attitudes and thoughts about the use of coercion in psychiatric acute wards, submitted). Ward variables consist of data about the organization, staff attitudes to coercion, staff to bed ratio and whether the ward was in an urban or rural setting. Ward level variables are shown in Table 2.

**Table 2:** Sample characteristics, ward variables

Ward variables:	
Acute wards	28

Admission wards	4

Mean number of beds	11 (SD = 3.5)

Mean staff to bed ratio	3.5 (SD = 0.8)

Wards in urban area^a^	8

Wards in rural area	24

Staff Attitude to Coercion Scale^b^	

Coercion as offending (mean, SD)	2.86 (SD = .24)

Coercion as care & security (mean, SD)	4.21 (SD = 1.6)

Coercion as treatment (mean, SD)	2.45 (SD = .21)

^a^Ward in city with more than 100 000 inhabitants

^b^Scale from 1 to 5 with higher ratings for greater agreement with attitude (mean score for wards)

### Staff Attitude to Coercion Scale (SACS)

The Staff Attitude to Coercion Scale is a questionnaire developed to measure staff attitudes and thoughts about the use of coercion in mental health care. The questionnaire was previously tested in two different samples, showing fairly good and stable psychometric properties. The internal consistency (Cronbach's alpha) of the subscales is 0.69-0.73. Additional psychometric properties were presented in a previous study [47]. The 15-item questionnaire is scored on a 5-point Likert scale. Mean values for SACS scores in this sample are shown in Table 2. The three subscales represent three different clusters of staff attitudes and are named as follows.

#### I. Coercion as offensive (critical attitude)

This view sees coercion as offensive towards patients. The dimension consists of six items reflecting the most critical attitudes to the use of coercion and focuses on a wish to reduce the use of coercion. Other aspects include that coercion is potentially harmful and offensive towards patients and can violate the relationship between caregiver and patient.

#### II. Coercion as care and security (pragmatic attitude)

This view sees coercion as needed for care and security. The dimension consists of six items that focus on the use of coercion for security reasons, and the opinion that using coercion is perceived as giving care. This attitude can be considered as being a middle position and has a pragmatic view on the use of coercion.

#### III. Coercion as treatment (positive attitude)

This view sees coercion as a treatment intervention. The dimension consists of three items reflecting the most positive view on the use of coercion. These items claim that the use of coercion is needed when patients lack insight into their own illness and that more coercion should be used.

### Statistical analysis

Health services research regularly involves questions where individual outcomes are influenced by contextual factors, such as that patient outcomes may be influenced by ward characteristics. Hence, explanatory variables may be defined at both the individual and contextual levels. Analytically, this raises some important methodological challenges. Standard statistical tests lean on the assumption of independence between observations, which is obviously not true if the context is an important factor. If this assumption is violated, estimates of the standard errors may be too narrow. Further on, the causal process affecting the probability of the outcome is likely to be affected both by individual and shared contextual factors such as patients within wards. The multilevel framework allows for simultaneous analysis of both individual and contextual variables and also takes into account the clustering structure of data [48].

The sample comprised two hierarchical levels (patients and wards), and the dependent variables had two values (0 = no use and 1 = use). Multilevel logistic regression in Stata was applied [48]. For the present analysis, this framework allowed the estimation of the relationship between coercion use and patient and ward level characteristics (fixed parameters), and the estimation of variance in coercion probability between wards that was not accounted for by individual and ward level factors (random parameters). The variance attributable to the ward level was estimated with the *intraclass correlation coefficient *(ICC). The ICC in multilevel logistic regression was estimated by the procedure presented by Snijders and Boskers [49], where *U*_*j *_in the equation is the between-ward variance:

Because the patients have been at risk of coercion for different lengths of time, the multivariable analysis is adjusted for patients' length of stay on ward (LOS) and LOS^2 ^to take nonlinearity into account.

## Results

### Differences in use of seclusion, restraint and involuntary medication among wards

**Figure 1:**
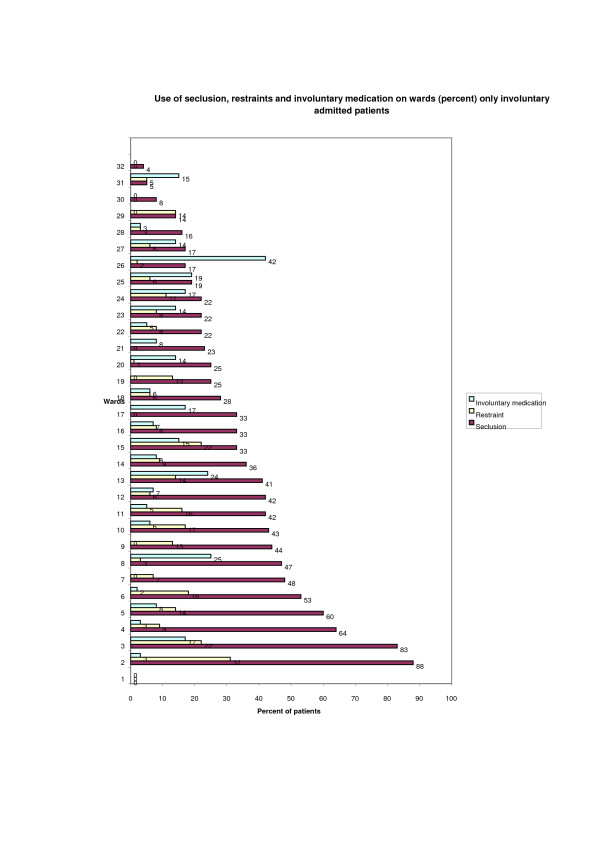
**Differences in the use of seclusion, restraint and involuntary medication among wards (*n *= 1214)**.

The total number of involuntary admitted patients was 1214 (35% of the total sample). The percentage of patients who were exposed to coercion ranged from 0-88% across wards. Of these patients, 424 (35%) had been secluded, 117 (10%) had been restrained and 113 (9%) patients had received involuntary depot medication at discharge. A total of 106 (9%) patients had been exposed to seclusion and restraint, 47 (4%) patients to seclusion and involuntary depot medication at discharge and 14 (1%) of patients to restraint and involuntary depot medication. A total of 13 (1%) patients had been exposed to all three forms of coercion. A diagram of the differences among the 32 wards in the use of the three coercive measures is shown in Figure 1.

There were data on all independent variables for 1016 patients and these were included in the multilevel logistic regression analysis (Additional file 1: Table S1).

### Seclusion

In a model adjusting only for LOS and LOS^2^, the ICC for the use of seclusion was 0.22. After adjustment for patient and ward level variables, the ICC for seclusion was reduced to 0.09 (*P *< .01). There was no statistically significant difference between male and female patients in the use of seclusion. There was a positive association between the risk of being secluded and aggressive/overactive, self-injury/suicidal and hallucinations/delusional symptoms, and there was a negative association between depressed mood and seclusion. There were no statistically significant associations between seclusion and drinking/drug problems, cognitive problems and physical illness. The differences in the risk of being secluded were small and not statistically significant among patients who were homeless or not, well known to referring agency or not, being intoxicated at admission or not and Norwegian or not. Wards in urban areas used seclusion more often (OR = 7.65) than wards in smaller towns and rural areas. There was a substantially lower level of patient seclusion in admission wards (OR = 0.19) compared with other ward types. The staff to bed ratio was not substantially associated with the use of seclusion, neither were ward means on the 3 SACS subscales.

### Restraint

For restraint, in a model adjusting only for LOS and LOS^2^, the ICC was 0.11 and statistically significant (*P *< .01). After adjustment for patient and ward level variables, the between-ward variance was reduced and not statistically significant. There was no substantial difference between male and female patients in the use of restraint. Based on assessment of psychiatric problems (HoNOS), there was a positive association between the risk of being restrained and aggressive/overactive and self-injury/suicidal symptoms. The other HoNOS variables were marginally associated with the risk of being restrained and not statistically significant. Patients from ethnic groups other than Norwegian had a lower risk of being restrained (OR = 0.39). The differences in the risk of being restrained were small and not statistically significant among patients being homeless or not and under the influence of drugs at admission or not. Wards in urban areas used restraint more often (OR = 3.58) than wards in smaller towns and rural areas. Admission wards were not statistically different from other wards in the use of restraint, neither did staff to bed ratio show any substantial influence. The associations among ward means for the 3 SACS scales and the use of restraint were not statistically significant.

### Involuntary medication

In a model adjusting only for LOS and LOS^2^, the ICC for use of involuntary medication was 0.20. Adjustment for individual and ward level variables reduced the ICC to 0.17 (*P *< .01). There was no substantial difference between male and female patients in the use of involuntary medication. Patients diagnosed with schizophrenia had a higher risk of being given involuntary medication (OR = 10.85) compared with patients in other diagnostic categories. None of the HoNOS variables was substantially associated with the risk of being medicated involuntarily. Patients known to the referring agency had a higher risk of being involuntarily medicated (OR = 3.27) compared with less known patients. Differences in the risk of being involuntarily medicated were small and not statistically significant among patients who were homeless or not, under the influence of drugs at admission or not and Norwegian or not. None of the ward variables was associated with the involuntary use of medication.

## Discussion

### Differences among wards in use of seclusion, restraint and involuntary medication

This cross-sectional observational national study showed substantial differences between Norwegian acute psychiatric wards concerning the use of seclusion, restraint and involuntary medication; however, this was influenced to some extent by compositional differences across wards, especially for the use of restraint. Several previous studies have reported substantial differences between treatment units regarding the use of coercion [12-14,18,19,21,22,50-53]. Nevertheless, this is the first study we have seen using a multi level approach analyzing both ward and patient characteristics as risk factors for the use of seclusion, restraint and involuntary medication.

### Patient characteristics

Patients with a diagnosis of schizophrenia or other psychosis have a substantially higher risk of being involuntarily medicated. This may be because this group of patients is the main group treated with involuntary medication. Some patients receiving involuntary treatment in the community are admitted to the hospital for the purpose of reinstalling depot medication after they have stopped taking the medication. A previous study showed that having received a diagnosis of schizophrenia, involuntary legal status and having been committed previously for treatment predicted the use of involuntary medication [16]. Patients who are overactive and aggressive, experiencing hallucinations and delusions, executing self-injury or at risk of suicide have a higher risk of being secluded and restrained than patients not showing such behaviour. The finding that overactivity and aggressiveness in patients most strongly predicts the use of seclusion and restraint indicates that this behaviour is a challenge for staff and often the reason for using coercive interventions. Patient aggressiveness as a main reason for using seclusion has also been found in other studies [52,54-56]. Reasons for patients' aggression and patient-staff interactions should be analysed and targeted for intervention to reduce the use of seclusion on wards [55,57]. A study by Keski-Valkama et al. found that of all of the patient characteristics they investigated, only main diagnosis and phase of stay were independent risk factors for restraint and seclusion [58].  They also concluded that to reduce the use of seclusion and restraint, resources should be targeted especially towards the most disturbed patients.

### Ward characteristics

Wards located in urban areas showed higher levels of seclusion and restraint compared with wards in rural areas and smaller towns. This may indicate that patients in urban areas have a greater number and range of problems. Furthermore, there may be more problems with drug use, homelessness and lack of social networks. Another possible explanation is that in hospitals in urban areas, patients are less well known to referring agencies.

A substantial portion of the differences in the use of coercive measures can be attributed to the ward level. To estimate the variance attributable to the ward level, we computed intraclass correlation coefficients (ICC) as a measure of how similar the wards were in their use of coercive measures. In a logistic regression analysis, we do not have information about the residuals in the same way as in a linear regression. Therefore, a calculation of explained variance is not available. However, the ICC as applied here may be understood as an estimate of the relative proportion of the variance represented by the ward level. The ICC value is largest for the use of involuntary medication. However, none of the ward variables that we entered in the equation predicted the use of involuntary medication. This finding indicates that there are ward characteristics other than those measured in our study that represent ward effects on the dependent variables (or that we did not assess ward-specific characteristics well enough). Future research should attempt to identify these characteristics. The fact that the ward level is an important influence on both the use of seclusion and involuntary medication may indicate that interventions regarding the use of coercive measures should take into account organizational factors. Furthermore, patient aggressiveness should be considered to be a product of staff-patient interaction and not only a trait or state of the patient. A review of the literature on interventions to reduce the use of seclusion gives support for complex interventions involving change to several aspects of the organization [59]. Another study also found that being in a hospital with high rates of seclusion and restraint resulted in higher risks of being secluded or restrained again [21].

### Staff attitudes and thoughts about use of coercion in mental health care

The three dimensions of staff attitudes towards the use of coercion were not substantially associated with the use of coercion in this study, contrary to the hypothesis. An explanation for this finding may be that staff attitudes do not predict differences in the use of coercion. That is, coercion is used regardless of staff attitudes. However, in this study, staff attitudes were aggregated on the ward level and expressed as staff group means. It could be that individual differences in attitudes influence the use of coercion, but that these individual differences are masked (hidden) when using group means for the staff. The staff groups may also be influenced by leaders or other persons acting as role models [60]. A third possible explanation could be methodological weaknesses with the instrument in the sense that the SACS scale might be unsuccessful in capturing relevant staff attitudes. Differences in ward culture and staff attitudes are still among the factors often mentioned as possible explanations for differences in the use of coercion [12,12,16,21,28,43], and should be investigated more thoroughly.

## Conclusions

The substantial between-ward variance even when adjusting for patients' individual psychopathology indicates a potential for quality improvement regarding the use of coercion. Hence, interventions to reduce coercion should take into account organizational and environmental factors and not only factors at the individual level. The results also indicate that to reduce the use of coercion, there should be a focus on interventions to reduce patients' aggressiveness and on addressing the special circumstances and needs of wards in urban areas. Interventions to reduce patients' aggressiveness may include increased empowerment for service users and user involvement. Staff training in communication and dialogue skills may also be effective in reducing conflict and moderating aggression. Future research should focus on staff-patient interaction, reasons for patient aggressiveness, how to meet patients' needs to avoid aggressive reactions and interventions to reduce the use of coercion in mental health care.

## Competing interests

The authors declare that they have no competing interests.

## Authors' contributions

TLH have made the questionnaire about the staff attitudes and thought about the use of coercive measures in mental health care (SACS) which is basis for some of these results, is main author for this manuscript and have performed descriptive statistics. JHB has performed the multilevel analysis and have participated in analysing and written out the results of the analysis. AF has been supervisor in the project and has participated in making the SACS questionnaire and in analyzing the results. TR has been head supervisor and project leader of the Multicenter study of Acute Psychiatry (MAP) and have participated in analyzing the results. All authors have read and approved the final manuscript.

## Pre-publication history

The pre-publication history for this paper can be accessed here:

http://www.biomedcentral.com/1472-6963/10/89/prepub

## Supplementary Material

Additional file 1 **Table S1: Multilevel logistic regression (Odds Ratio), only involuntary admitted patients in the analysis**. Results of the multilevel logistic regression analysis (patient and ward variables). Click here for file
