# Supplementary material for: A cross-sectional prospective study of seclusion, restraint and involuntary medication in acute psychiatric wards: patient, staff and ward characteristics
Source: BMC Health Serv Res. 2010 Apr 6;10:89. doi: 10.1186/1472-6963-10-89 (PMC2858144; doi:10.1186/1472-6963-10-89)
Supplement: Additional file 1 — Table S1: Multilevel logistic regression (Odds Ratio), only involuntary admitted patients in the analysis. Results of the multilevel logistic regression analysis (patient and ward variables). [file 1472-6963-10-89-S1.DOC]

|  | **Seclusion** | | **Restraint** | | **Involuntary medication** | |
| --- | --- | --- | --- | --- | --- | --- |
| ***Patient variables*** | Odds Ratio | 95% Conf. Int. | Odds Ratio | 95% Conf. Int. | Odds Ratio | 95% Conf. Int. |
| Sex (female) | 1.29 | .92–1.79 | .67 | .39–1.13 | .98 | .57–1.67 |
| Other than Norwegian | 1.15 | .70–1.88 | .39* | .16–.96 | .50 | .20–1.24 |
| Not having own home | 1.85 | .89–3.81 | 1.34 | .49–3.64 | .44 | .09–2.13 |
| Patient known to referring agency | .80 | .57–1.13 | .57 | .32–1.02 | 3.27*** | 1.87–5.71 |
| F 20–29 diagnosis (ICD–10) | 1.04 | .71–1.51 | 1.76 | .99–3.14 | 10.85*** | 5.32–22.13 |
| Intoxicated at admission | 1.48 | .85–2.56 | 1.43 | .65–3.12 | .79 | .30–2.08 |
| HoNOS 1 (overactive & aggressive) | 1.88*** | 1.65–2.15 | 2.38*** | 1.91–2.98 | 1.07 | .86–1.33 |
| HoNOS 2 (self-injury & suicidal) | 1.18* | 1.02–1.37 | 1.39** | 1.12–1.73 | 1.02 | .75–1.39 |
| HoNOS 3 (drinking & drugs) | .89 | .76–1.04 | .96 | .76–1.21 | .98 | .75–1.27 |
| HoNOS 4 (cognitive problems) | 1.11 | .97–1.27 | .97 | .78–1.20 | 1.02 | .81–1.29 |
| HoNOS 5 (physical illness & disability) | .94 | .80–1.10 | .95 | .73–1.24 | .88 | .68–1.15 |
| HoNOS 6 (hallucinations & delusions) | 1.17* | 1.02–1.33 | 1.00 | .81–1.24 | .96 | .76–1.20 |
| HoNOS 7 (depressed mood) | .83* | .71–.98 | 1.05 | .81–1.35 | .82 | .62–1.07 |
| *Ward variables* |  |  |  |  |  |  |
| Admission ward | .19*** | .08–.46 | .53 | .26–1.16 | .80 | .16–4.03 |
| Staff to bed ratio | 1.37 | .91–2.07 | 1.58 | 1.07–2.66 | 1.14 | .59–2.20 |
| Ward in urban area | 7.65*** | 3.36–17.49 | 3.58** | 1.28–4.86 | .48 | .11–2.03 |
| SACS: Offending staff attitude (mean) | 1.23 | .36–4.20 | .35 | .11–1.16 | 4.04 | .47–34.63 |
| SACS: Security staff attitude (mean) | 4.04 | .72–22.83 | .99 | .07–3.11 | 1.11 | .06–20.48 |
| SACS: Treatment staff attitude (mean) | 1.14 | .30–4.35 | 1.93 | .46–11.48 | .86 | .08–9.53 |
| Between-ward variance | .32*** | .11–.88 | .04 | .00–30.50 | .68*** | .24–1.91 |
| ICC | .09 |  | .01 |  | .17 |  |
| *n* | 1016 |  | 1016 |  | 1016 |  |

**p* < .05

***p* < .01

*** *p* < .000
